# 3-Oxo-18α-olean-28,13β-olide

**DOI:** 10.1107/S160053681002903X

**Published:** 2010-07-31

**Authors:** R. C. Santos, R. M. A. Pinto, A. Matos Beja, J. A. R. Salvador, J. A. Paixão

**Affiliations:** a Laboratório de Química Farmacêutica, Faculdade de Farmácia, Universidade de Coimbra, Pólo das Ciências da Saúde, Azinhaga de Santa Comba, P-3000-548 Coimbra, Portugal; b CEMDRX, Departamento de Física, Faculdade de Ciências e Tecnologia, Universidade de Coimbra, P-3004-516 Coimbra, Portugal

## Abstract

The title terpene, C_30_H_46_O_3_, is a 28,13β-lactone of oleanolic acid prepared with bis­muth trifluoro­methane­sulfonate (O*Tf*), Bi(O*Tf*)_3_·*x*H_2_O. All rings are *trans*-fused. The X-ray study shows the inversion of the orientation of 18-*H* in the lactonization reaction. A quantum chemical *ab*-*initio* Roothaan Hartree–Fock calculation of the equilibrium geometry of the isolated mol­ecule gives values for bond lengths and valency angles in close agreement with experimental values. The calculation also reproduces the observed mol­ecular conformation, with puckering parameters that agree well with those determined from the crystallographic study.

## Related literature

For general background to the use of natural products as sources of anti­cancer drugs, see: Koehn & Carter (2005 ▶). For the biological activity of oleanolic acid, see: Ringbom *et al.* (1998 ▶); Ma *et al.* (2000 ▶); Tokuda *et al.* (1986 ▶); Horiuchi *et al.* (2007 ▶); Lee *et al.* (1994 ▶); Sohn *et al.* (1995 ▶). For the bio­syn­thesis of penta­cyclic triterpenoids, see: Gershenzon & Dudareva (2007 ▶); Salvador (2010 ▶); Dzubak *et al.* (2006 ▶). For the lactonization reaction of oleanane-type triterpenoids, see: Cheriti *et al.* (1994 ▶). For the synthesis of the title compound, see: Salvador *et al.* (2009 ▶). For related structures, see: Eggleston (1987 ▶); Chang *et al.* (1982 ▶); Sutthivaiyakit *et al.* (2001 ▶); Wang *et al.* (2006 ▶). For puckering and asymmetry parameters, see: Cremer & Pople (1975 ▶); Duax & Norton (1975 ▶). The quantum chemical calculations were performed with the computer program *GAMESS* (Schmidt *et al.*, 1993 ▶).
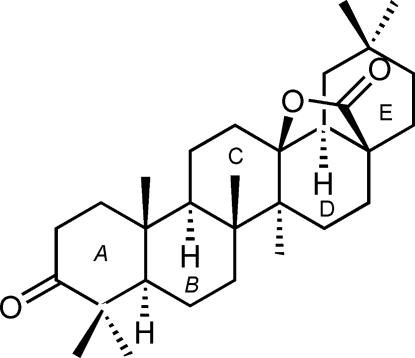


## Experimental

### 

#### Crystal data

C_30_H_46_O_3_
*M* *_r_* = 454.67
Monoclinic, 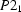

*a* = 6.7789 (3) Å
*b* = 12.3122 (6) Å
*c* = 15.4524 (7) Å
β = 99.644 (2)°
*V* = 1271.48 (10) Å^3^
*Z* = 2
Mo *K*α radiation
μ = 0.07 mm^−1^
*T* = 295 K
0.45 × 0.17 × 0.04 mm

#### Data collection

Bruker APEXII CCD area-detector diffractometer
Absorption correction: multi-scan (*SADABS*; Sheldrick, 2000 ▶) *T*_min_ = 0.746, *T*_max_ = 1.0
16467 measured reflections
2536 independent reflections
1805 reflections with *I* > 2σ(*I*)
*R*_int_ = 0.057

#### Refinement

*R*[*F* ^2^ > 2σ(*F* ^2^)] = 0.042
*wR*(*F* ^2^) = 0.100
*S* = 1.08
2536 reflections
305 parameters
1 restraint
H-atom parameters constrained
Δρ_max_ = 0.14 e Å^−3^
Δρ_min_ = −0.17 e Å^−3^

### 

Data collection: *APEX2* (Bruker, 2006 ▶); cell refinement: *SAINT* (Bruker, 2006 ▶); data reduction: *SAINT*; program(s) used to solve structure: *SHELXS97* (Sheldrick, 2008 ▶); program(s) used to refine structure: *SHELXL97* (Sheldrick, 2008 ▶); molecular graphics: *PLATON* (Spek, 2009 ▶); software used to prepare material for publication: *SHELXL97*.

## Supplementary Material

Crystal structure: contains datablocks global, I. DOI: 10.1107/S160053681002903X/rk2222sup1.cif

Structure factors: contains datablocks I. DOI: 10.1107/S160053681002903X/rk2222Isup2.hkl

Additional supplementary materials:  crystallographic information; 3D view; checkCIF report

## Figures and Tables

**Fig. 1.**
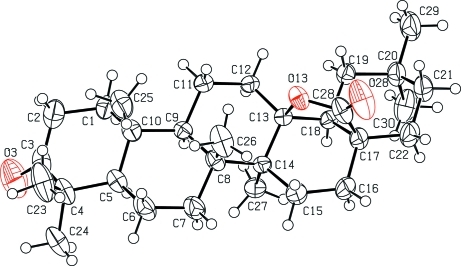
Molecular structure of the title compound with the atom numbering scheme. Displacement ellipsoids are drawn at the 50% probability level. The H atoms are presented as a small spheres of arbitrary radius.

## supplementary crystallographic information

### Comment

The natural products have been the source of the main anticancer drugs for centuries and represent 50% of drugs used in the clinic in developed countries (Koehn & Carter, 2005). As the largest class of natural products, pentacyclic triterpenoids biosynthesized in plants by squalene cyclization represent a varied class of bioactive natural products (Gershenzon & Dudareva, 2007; Salvador, 2010; Dzubak *et al.*, 2006). Among them oleanolic acid was reported to display several biological effects including anti-inflammatory (Ringbom *et al.*, 1998), anti-viral (Ma *et al.*, 2000), anti-bacterial (Horiuchi *et al.*, 2007) and in particular anti-cancer activities. It has been shown to act at various stages of tumor development, including inhibition of tumourigenesis, inhibition of tumor promotion (Tokuda *et al.*, 1986), induction of tumor cell differentiation and apoptosis (Lee *et al.*, 1994) and inhibition of angiogenesis, invasion tumor cells and metastasis (Sohn *et al.*, 1995). The lactonization reaction of oleanane type triterpenoids, with a C12═C13 double bond, under acid conditions has been reported. This classical transformation involves a 28,13β-lactonization with 18-*H* inversion of orientation with the formation of an oleanane type γ-lactone (Cheriti *et al.*, 1994). As part of our current interest on the application of bismuth(III) salts to the chemistry of triterpenoids (Salvador *et al.*, 2009), we have recently reported the 28,13β-lactonization of oleanolic acid in CH_2_Cl_2_, using bismuth trifluoromethanesulfonate, Bi(O*Tf*)_3_^.^*x*H_2_O (Salvador *et al.*, 2009). Mindful of the biological and synthetic importance of such molecules, we report in this communication the molecular structure of the 3-oxo-18α-olean-28,13β-olide determined by single-crystal X-ray diffraction, and compare it with that of the free molecule as given by quantum mechanical *ab-initio* calculation.

The structure of this compound with the corresponding atomic numbering scheme is shown in Fig. 1. This triterpenoid compound is an oleanane type with a 28,13β-lactone. The typical C12═C13 double bond is absent. The inversion of orientation of 18-*H* in the lactonization reaction was unequivocally demonstrated by this X-ray crystallographic study. Bond lengths and angles are within the range of expected average values. All six-membered rings are fused *trans*- and have slightly distorted chair conformations, the D-ring being more heavily distorted towards a half-chair conformation due to the strain induced by the lactonization, as shown by the Cremer & Pople, (1975) parameters: [ring A: Q = 0.517 (4)Å, θ = 6.8 (4)° and φ = 341 (4)°; B: Q = 0.570 (3)Å, θ = 11.7 (3)° and φ = 3.9 (17)°; C: Q = 0.573 (3)Å, θ = 12.0 (3)° and φ = 23.8 (14)°; D: Q = 0.646 (3)Å, θ = 20.5 (3)° and φ = 65.3 (9)°; E: Q = 0.522 (4)Å, θ = 12.8 (4)° and φ = 181.5 (17)°].

The lactone ring has an envelope conformation [q_2_ = 0.457 (3)Å and φ_2_ = 71.6 (4)° and asymmetry parameters (Duax & Norton, 1975) ΔC_s_(C18) = ΔC_s_(C28, O13) = 0.8 (3)°].

*Ab*-*initio* Roothaan Hartree–Fock calculations reproduce well the observed bond length and valency angles of the molecule. Also, the calculated conformation of the rings are very close to the experimental values.

There are no strong hydrogen bonds in the crystal structure, due to the lack of strong H-donors. One weak C—H···O intramolecular interaction can be spotted in the molecule, involving atoms C26 and O13.

### Experimental

To a solution of oleanonic acid (91.4 mg, 0.20 mmol) in CH_2_Cl_2_ (10 ml), Bi(O*Tf*)_3_^.^*x*H_2_O (29.1 mg, 0.04 mmol) was added. After 24 h under magnetic stirring at reflux temperature, the reaction was completed as verified by *TLC* control. The reaction mixture was concentrated under reduced pressure and the resulting residue dissolved in diethyl ether (100 ml). The organic phase was washed with NaHCO_3_ (10% aq), water, dried with anhydrous Na_2_SO_4_, and concentrated under reduced pressure to give the title compound as a white solid (86.8 mg, 95% yield). M.p. with thermal decomposition observed at about 583 K (from acetonitrile/acetone); IR (film) 2958, 1757, 1703, 1447, 1393, 1260 cm^-1^; ^1^H NMR (400 MHz; CDCl_3_; *Me*_4_Si) 0.84 (3 H, s), 0.89 (3 H, s), 1.00 (3 H, s), 1.04 (3 H, s), 1.09 (3 H, s), 1.15 (3 H, d, *J* 1/2), 1.22 (3 H, s), 2.41 (1 H, td, *J* 15.8, 7.3 and 4.2, 2-H_a_), 2.54 (1 H, td, *J* 15.8, 10.3 and 7.5, 2-H_b_); ^13^C NMR (100 MHz; CDCl_3_; *Me*_4_Si) 16.0, 17.8, 18.7, 19.1, 19.4, 21.1, 23.1, 26.0, 26.5, 26.6, 27.8, 29.9, 31.5, 33.0, 34.1, 34.2, 35.2, 36.2, 36.7, 39.8, 41.3, 43.9, 45.0, 47.3, 47.4, 49.4, 54.9, 89.5, 179.1, 217.6; EI–MS m/*z* (%): 455 (18) [*M*+ 1]^+^, 437 (5), 409 (4), 235 (15), 218 (38), 203 (64), 189 (100), 119 (93).

In order to gain some insight on how the crystal packing of title compound might affect the molecular geometry we have performed a quantum chemical calculation on the equilibrium geometry of the free molecule. These calculations were performed with the computer program GAMESS (Schmidt *et al.*, 1993). A molecular orbital Roothan Hartree–Fock method was used with an extended 6-31 G(d,p) basis set. Tight conditions for convergence of both the self-consistent field cycles and maximum density and energy gradient variations were imposed (10^-6^ atomic units). The program was run on the Milipeia cluster of UC–LCA (using 16 Opteron cores, 2.2 GHz runing Linux).

### Refinement

All hydrogen atoms were refined as riding on their parent atoms using *SHELXL97* defaults: C—H = 0.97Å with *U*_iso_(H) = 1.2*U*_eq_(C) for methylene H; C—H = 0.96Å with *U*_iso_(H) = 1.5*U*_eq_(C) for methyl H; C—H = 0.98Å with *U*_iso_(H) = 1.2*U*_eq_(C) for methine H. The absolute configuration was not determined from the X-ray data, as the molecule lacks any strong anomalous scatterer atom at the Mo Kα wavelength, but was known from the synthetic route. Friedel pairs of reflections (2247 pairs) were merged before refinement.

### Crystal data

**Table:**

C_30_H_46_O_3_	*D*_x_ = 1.188 Mg m^−^^3^
*M**_r_* = 454.67	Melting point: 583 K
Monoclinic, *P*2_1_	Mo *K*α radiation, λ = 0.71073 Å
*a* = 6.7789 (3) Å	Cell parameters from 4019 reflections
*b* = 12.3122 (6) Å	θ = 3.1–21.5°
*c* = 15.4524 (7) Å	µ = 0.07 mm^−^^1^
β = 99.644 (2)°	*T* = 295 K
*V* = 1271.48 (10) Å^3^	Plate, colourless
*Z* = 2	0.45 × 0.17 × 0.04 mm
*F*(000) = 500	

### Data collection

**Table:**

Bruker APEXII CCD area-detector diffractometer	2536 independent reflections
Radiation source: fine-focus sealed tube	1805 reflections with *I* > 2σ(*I*)
graphite	*R*_int_ = 0.057
φ– and ω–scans	θ_max_ = 25.8°, θ_min_ = 3.3°
Absorption correction: multi-scan (*SADABS*; Sheldrick, 2000)	*h* = −8→8
*T*_min_ = 0.746, *T*_max_ = 1.0	*k* = −14→15
16467 measured reflections	*l* = −18→18

### Refinement

**Table:**

Refinement on *F*^2^	Primary atom site location: structure-invariant direct methods
Least-squares matrix: full	Secondary atom site location: difference Fourier map
*R*[*F*^2^ > 2σ(*F*^2^)] = 0.042	Hydrogen site location: inferred from neighbouring sites
*wR*(*F*^2^) = 0.100	H-atom parameters constrained
*S* = 1.08	*w* = 1/[σ^2^(*F*_o_^2^) + (0.0386*P*)^2^ + 0.2321*P*] where *P* = (*F*_o_^2^ + 2*F*_c_^2^)/3
2536 reflections	(Δ/σ)_max_ < 0.001
305 parameters	Δρ_max_ = 0.14 e Å^−^^3^
1 restraint	Δρ_min_ = −0.17 e Å^−^^3^

### Special details

**Table:**

Geometry. All s.u.'s (except the s.u. in the dihedral angle between two l.s. planes) are estimated using the full covariance matrix. The cell s.u.'s are taken into account individually in the estimation of s.u.'s in distances, angles and torsion angles; correlations between s.u.'s in cell parameters are only used when they are defined by crystal symmetry. An approximate (isotropic) treatment of cell s.u.'s is used for estimating s.u.'s involving l.s. planes.
Refinement. Refinement of *F*^2^ against ALL reflections. The weighted *R*-factor *wR* and goodness of fit *S* are based on *F*^2^, conventional *R*-factors *R* are based on *F*, with *F* set to zero for negative *F*^2^. The threshold expression of *F*^2^ > σ(*F*^2^) is used only for calculating *R*-factors(gt) *etc*. and is not relevant to the choice of reflections for refinement. *R*-factors based on *F*^2^ are statistically about twice as large as those based on *F*, and *R*-factors based on ALL data will be even larger.

### Fractional atomic coordinates and isotropic or equivalent isotropic displacement parameters (Å^2^)

**Table:**

	*x*	*y*	*z*	*U*_iso_*/*U*_eq_	
O13	0.4607 (3)	−0.02601 (19)	0.48624 (13)	0.0397 (6)	
O28	0.7505 (3)	0.0179 (3)	0.56915 (16)	0.0794 (10)	
O3	−0.5087 (4)	−0.0157 (4)	0.0038 (2)	0.0996 (12)	
C1	−0.1906 (5)	−0.1242 (3)	0.1826 (2)	0.0491 (9)	
H1A	−0.2856	−0.0918	0.2154	0.059*	
H1B	−0.1540	−0.1948	0.2081	0.059*	
C2	−0.2931 (6)	−0.1399 (4)	0.0868 (2)	0.0646 (11)	
H2A	−0.2056	−0.1812	0.0554	0.077*	
H2B	−0.4155	−0.1812	0.0856	0.077*	
C3	−0.3412 (5)	−0.0339 (4)	0.0419 (2)	0.0575 (10)	
C4	−0.1742 (5)	0.0484 (3)	0.0468 (2)	0.0520 (10)	
C23	−0.0394 (6)	0.0166 (5)	−0.0194 (2)	0.0835 (16)	
H23A	−0.1123	0.0244	−0.0780	0.125*	
H23B	0.0026	−0.0575	−0.0098	0.125*	
H23C	0.0760	0.0631	−0.0120	0.125*	
C24	−0.2693 (7)	0.1595 (4)	0.0201 (3)	0.0878 (16)	
H24A	−0.3445	0.1550	−0.0382	0.132*	
H24B	−0.1660	0.2131	0.0217	0.132*	
H24C	−0.3568	0.1796	0.0603	0.132*	
C5	−0.0633 (4)	0.0555 (3)	0.1440 (2)	0.0407 (8)	
H5	−0.1623	0.0875	0.1760	0.049*	
C6	0.1103 (5)	0.1352 (3)	0.1581 (2)	0.0583 (11)	
H6A	0.0742	0.2001	0.1234	0.070*	
H6B	0.2257	0.1027	0.1387	0.070*	
C7	0.1632 (5)	0.1656 (3)	0.2547 (2)	0.0542 (10)	
H7A	0.0514	0.2047	0.2717	0.065*	
H7B	0.2770	0.2144	0.2621	0.065*	
C8	0.2137 (4)	0.0679 (3)	0.3170 (2)	0.0356 (8)	
C26	0.4243 (4)	0.0295 (4)	0.3042 (2)	0.0578 (11)	
H26A	0.4297	0.0250	0.2426	0.087*	
H26B	0.4510	−0.0407	0.3306	0.087*	
H26C	0.5229	0.0804	0.3315	0.087*	
C9	0.0551 (4)	−0.0227 (2)	0.29225 (17)	0.0292 (7)	
H9	−0.0679	0.0065	0.3088	0.035*	
C10	−0.0033 (4)	−0.0528 (3)	0.19274 (19)	0.0376 (8)	
C11	0.1095 (5)	−0.1204 (3)	0.3527 (2)	0.0406 (8)	
H11A	0.0099	−0.1769	0.3373	0.049*	
H11B	0.2376	−0.1491	0.3435	0.049*	
C12	0.1212 (4)	−0.0914 (2)	0.44899 (19)	0.0335 (7)	
H12A	−0.0135	−0.0785	0.4601	0.040*	
H12B	0.1738	−0.1536	0.4840	0.040*	
C13	0.2476 (4)	0.0060 (2)	0.48011 (18)	0.0266 (7)	
C14	0.2075 (4)	0.1043 (2)	0.4157 (2)	0.0317 (7)	
C27	−0.0006 (4)	0.1511 (3)	0.4245 (2)	0.0460 (9)	
H27A	−0.0924	0.0924	0.4278	0.069*	
H27B	−0.0491	0.1954	0.3743	0.069*	
H27C	0.0108	0.1943	0.4768	0.069*	
C15	0.3649 (5)	0.1931 (3)	0.4452 (2)	0.0546 (10)	
H15A	0.4879	0.1741	0.4246	0.066*	
H15B	0.3177	0.2612	0.4177	0.066*	
C16	0.4105 (6)	0.2097 (3)	0.5445 (2)	0.0559 (11)	
H16A	0.3018	0.2502	0.5628	0.067*	
H16B	0.5313	0.2528	0.5590	0.067*	
C17	0.4383 (4)	0.1023 (3)	0.5959 (2)	0.0376 (8)	
C22	0.5210 (5)	0.1223 (3)	0.6926 (2)	0.0504 (10)	
H22A	0.6595	0.1451	0.6982	0.061*	
H22B	0.4464	0.1809	0.7139	0.061*	
C21	0.5095 (5)	0.0229 (3)	0.7492 (2)	0.0498 (9)	
H21A	0.5475	0.0430	0.8104	0.060*	
H21B	0.6049	−0.0307	0.7358	0.060*	
C20	0.3009 (5)	−0.0285 (3)	0.7359 (2)	0.0436 (8)	
C19	0.2422 (4)	−0.0582 (3)	0.63857 (18)	0.0361 (8)	
H19A	0.3342	−0.1129	0.6240	0.043*	
H19B	0.1093	−0.0899	0.6293	0.043*	
C18	0.2434 (4)	0.0383 (2)	0.57641 (18)	0.0288 (7)	
H18	0.1292	0.0860	0.5797	0.035*	
C29	0.3103 (7)	−0.1316 (4)	0.7907 (2)	0.0714 (13)	
H29A	0.1787	−0.1622	0.7860	0.107*	
H29B	0.3608	−0.1147	0.8510	0.107*	
H29C	0.3975	−0.1831	0.7696	0.107*	
C30	0.1485 (5)	0.0488 (4)	0.7642 (2)	0.0659 (12)	
H30A	0.0215	0.0129	0.7596	0.099*	
H30B	0.1350	0.1117	0.7269	0.099*	
H30C	0.1929	0.0707	0.8239	0.099*	
C28	0.5721 (4)	0.0295 (3)	0.5524 (2)	0.0458 (9)	
C25	0.1610 (5)	−0.1171 (4)	0.1569 (2)	0.0607 (11)	
H25A	0.2562	−0.0674	0.1394	0.091*	
H25B	0.1014	−0.1594	0.1071	0.091*	
H25C	0.2277	−0.1645	0.2018	0.091*	

### Atomic displacement parameters (Å^2^)

**Table:**

	*U*^11^	*U*^22^	*U*^33^	*U*^12^	*U*^13^	*U*^23^
O13	0.0281 (10)	0.0565 (15)	0.0345 (12)	0.0119 (10)	0.0056 (9)	0.0031 (12)
O28	0.0242 (12)	0.156 (3)	0.0571 (16)	0.0006 (16)	0.0051 (10)	0.0102 (19)
O3	0.0579 (16)	0.149 (3)	0.082 (2)	−0.002 (2)	−0.0183 (14)	0.010 (2)
C1	0.061 (2)	0.047 (2)	0.037 (2)	−0.0057 (18)	0.0020 (16)	−0.0074 (18)
C2	0.067 (2)	0.071 (3)	0.051 (3)	−0.010 (2)	−0.0014 (19)	−0.013 (2)
C3	0.052 (2)	0.084 (3)	0.033 (2)	0.006 (2)	−0.0018 (16)	−0.009 (2)
C4	0.051 (2)	0.070 (3)	0.0314 (19)	0.007 (2)	−0.0031 (15)	0.0083 (19)
C23	0.070 (3)	0.152 (5)	0.030 (2)	0.003 (3)	0.0140 (18)	0.006 (3)
C24	0.106 (3)	0.093 (4)	0.051 (3)	0.016 (3)	−0.025 (2)	0.017 (3)
C5	0.0375 (16)	0.052 (2)	0.0326 (18)	0.0052 (16)	0.0044 (13)	0.0061 (16)
C6	0.064 (2)	0.069 (3)	0.039 (2)	−0.013 (2)	0.0010 (17)	0.021 (2)
C7	0.064 (2)	0.052 (2)	0.043 (2)	−0.0184 (19)	−0.0026 (17)	0.0150 (19)
C8	0.0326 (15)	0.043 (2)	0.0314 (18)	−0.0024 (14)	0.0055 (13)	0.0088 (15)
C26	0.0335 (16)	0.100 (3)	0.043 (2)	0.002 (2)	0.0128 (14)	0.005 (2)
C9	0.0298 (14)	0.0299 (18)	0.0292 (16)	0.0054 (13)	0.0086 (11)	0.0008 (15)
C10	0.0355 (16)	0.046 (2)	0.0319 (18)	0.0097 (15)	0.0074 (12)	−0.0008 (16)
C11	0.0567 (19)	0.0298 (18)	0.0341 (19)	0.0041 (16)	0.0036 (14)	−0.0011 (15)
C12	0.0444 (17)	0.0251 (17)	0.0308 (18)	−0.0017 (14)	0.0057 (13)	0.0006 (14)
C13	0.0239 (13)	0.0277 (17)	0.0291 (16)	0.0007 (12)	0.0072 (11)	0.0002 (13)
C14	0.0334 (15)	0.0265 (17)	0.0339 (18)	−0.0027 (13)	0.0021 (12)	0.0023 (15)
C27	0.0503 (19)	0.038 (2)	0.047 (2)	0.0142 (16)	−0.0005 (15)	−0.0047 (17)
C15	0.068 (2)	0.044 (2)	0.047 (2)	−0.0272 (19)	−0.0070 (17)	0.0108 (18)
C16	0.071 (2)	0.041 (2)	0.050 (2)	−0.0314 (19)	−0.0097 (18)	0.0028 (19)
C17	0.0334 (15)	0.043 (2)	0.0347 (18)	−0.0147 (14)	0.0018 (13)	0.0031 (16)
C22	0.0487 (19)	0.055 (3)	0.045 (2)	−0.0213 (18)	−0.0012 (15)	−0.0043 (19)
C21	0.0466 (18)	0.063 (2)	0.037 (2)	−0.0108 (18)	−0.0020 (14)	0.000 (2)
C20	0.0480 (17)	0.052 (2)	0.0299 (17)	−0.0110 (17)	0.0046 (13)	−0.0015 (18)
C19	0.0387 (16)	0.039 (2)	0.0314 (18)	−0.0067 (14)	0.0076 (12)	−0.0006 (15)
C18	0.0245 (13)	0.0292 (18)	0.0326 (17)	−0.0015 (12)	0.0044 (11)	−0.0013 (15)
C29	0.098 (3)	0.077 (3)	0.035 (2)	−0.030 (3)	−0.001 (2)	0.008 (2)
C30	0.059 (2)	0.094 (3)	0.048 (2)	−0.010 (2)	0.0200 (17)	−0.025 (2)
C28	0.0321 (17)	0.070 (3)	0.0352 (19)	−0.0053 (17)	0.0054 (13)	0.013 (2)
C25	0.062 (2)	0.081 (3)	0.038 (2)	0.033 (2)	0.0065 (17)	−0.006 (2)

### Geometric parameters (Å, °)

**Table:**

O13—C28	1.351 (4)	C11—H11B	0.9700
O13—C13	1.485 (3)	C12—C13	1.505 (4)
O28—C28	1.202 (3)	C12—H12A	0.9700
O3—C3	1.209 (4)	C12—H12B	0.9700
C1—C10	1.530 (4)	C13—C18	1.545 (4)
C1—C2	1.539 (5)	C13—C14	1.562 (4)
C1—H1A	0.9700	C14—C15	1.542 (4)
C1—H1B	0.9700	C14—C27	1.551 (4)
C2—C3	1.488 (6)	C27—H27A	0.9600
C2—H2A	0.9700	C27—H27B	0.9600
C2—H2B	0.9700	C27—H27C	0.9600
C3—C4	1.512 (5)	C15—C16	1.528 (5)
C4—C23	1.533 (5)	C15—H15A	0.9700
C4—C24	1.538 (6)	C15—H15B	0.9700
C4—C5	1.566 (4)	C16—C17	1.537 (5)
C23—H23A	0.9600	C16—H16A	0.9700
C23—H23B	0.9600	C16—H16B	0.9700
C23—H23C	0.9600	C17—C28	1.510 (5)
C24—H24A	0.9600	C17—C18	1.525 (4)
C24—H24B	0.9600	C17—C22	1.526 (4)
C24—H24C	0.9600	C22—C21	1.514 (5)
C5—C6	1.520 (5)	C22—H22A	0.9700
C5—C10	1.551 (4)	C22—H22B	0.9700
C5—H5	0.9800	C21—C20	1.531 (4)
C6—C7	1.523 (5)	C21—H21A	0.9700
C6—H6A	0.9700	C21—H21B	0.9700
C6—H6B	0.9700	C20—C30	1.521 (5)
C7—C8	1.542 (5)	C20—C29	1.522 (5)
C7—H7A	0.9700	C20—C19	1.534 (4)
C7—H7B	0.9700	C19—C18	1.528 (4)
C8—C26	1.548 (4)	C19—H19A	0.9700
C8—C9	1.552 (4)	C19—H19B	0.9700
C8—C14	1.597 (4)	C18—H18	0.9800
C26—H26A	0.9600	C29—H29A	0.9600
C26—H26B	0.9600	C29—H29B	0.9600
C26—H26C	0.9600	C29—H29C	0.9600
C9—C11	1.530 (4)	C30—H30A	0.9600
C9—C10	1.567 (4)	C30—H30B	0.9600
C9—H9	0.9800	C30—H30C	0.9600
C10—C25	1.544 (4)	C25—H25A	0.9600
C11—C12	1.520 (4)	C25—H25B	0.9600
C11—H11A	0.9700	C25—H25C	0.9600
			
C28—O13—C13	109.2 (2)	O13—C13—C12	107.7 (2)
C10—C1—C2	113.9 (3)	O13—C13—C18	100.44 (19)
C10—C1—H1A	108.8	C12—C13—C18	114.3 (2)
C2—C1—H1A	108.8	O13—C13—C14	108.1 (2)
C10—C1—H1B	108.8	C12—C13—C14	112.7 (2)
C2—C1—H1B	108.8	C18—C13—C14	112.6 (2)
H1A—C1—H1B	107.7	C15—C14—C27	107.8 (3)
C3—C2—C1	111.5 (3)	C15—C14—C13	108.9 (2)
C3—C2—H2A	109.3	C27—C14—C13	107.2 (2)
C1—C2—H2A	109.3	C15—C14—C8	110.7 (2)
C3—C2—H2B	109.3	C27—C14—C8	111.0 (2)
C1—C2—H2B	109.3	C13—C14—C8	111.2 (2)
H2A—C2—H2B	108.0	C14—C27—H27A	109.5
O3—C3—C2	120.3 (4)	C14—C27—H27B	109.5
O3—C3—C4	122.3 (4)	H27A—C27—H27B	109.5
C2—C3—C4	117.4 (3)	C14—C27—H27C	109.5
C3—C4—C23	108.7 (3)	H27A—C27—H27C	109.5
C3—C4—C24	107.8 (3)	H27B—C27—H27C	109.5
C23—C4—C24	108.5 (4)	C16—C15—C14	113.9 (3)
C3—C4—C5	108.7 (3)	C16—C15—H15A	108.8
C23—C4—C5	114.3 (3)	C14—C15—H15A	108.8
C24—C4—C5	108.7 (3)	C16—C15—H15B	108.8
C4—C23—H23A	109.5	C14—C15—H15B	108.8
C4—C23—H23B	109.5	H15A—C15—H15B	107.7
H23A—C23—H23B	109.5	C15—C16—C17	113.0 (3)
C4—C23—H23C	109.5	C15—C16—H16A	109.0
H23A—C23—H23C	109.5	C17—C16—H16A	109.0
H23B—C23—H23C	109.5	C15—C16—H16B	109.0
C4—C24—H24A	109.5	C17—C16—H16B	109.0
C4—C24—H24B	109.5	H16A—C16—H16B	107.8
H24A—C24—H24B	109.5	C28—C17—C18	99.8 (2)
C4—C24—H24C	109.5	C28—C17—C22	112.5 (3)
H24A—C24—H24C	109.5	C18—C17—C22	116.2 (3)
H24B—C24—H24C	109.5	C28—C17—C16	108.1 (3)
C6—C5—C10	110.6 (2)	C18—C17—C16	108.4 (2)
C6—C5—C4	114.1 (3)	C22—C17—C16	111.1 (3)
C10—C5—C4	117.5 (3)	C21—C22—C17	112.9 (3)
C6—C5—H5	104.3	C21—C22—H22A	109.0
C10—C5—H5	104.3	C17—C22—H22A	109.0
C4—C5—H5	104.3	C21—C22—H22B	109.0
C5—C6—C7	110.4 (3)	C17—C22—H22B	109.0
C5—C6—H6A	109.6	H22A—C22—H22B	107.8
C7—C6—H6A	109.6	C22—C21—C20	113.1 (3)
C5—C6—H6B	109.6	C22—C21—H21A	109.0
C7—C6—H6B	109.6	C20—C21—H21A	109.0
H6A—C6—H6B	108.1	C22—C21—H21B	109.0
C6—C7—C8	114.3 (3)	C20—C21—H21B	109.0
C6—C7—H7A	108.7	H21A—C21—H21B	107.8
C8—C7—H7A	108.7	C30—C20—C29	109.3 (3)
C6—C7—H7B	108.7	C30—C20—C21	111.1 (3)
C8—C7—H7B	108.7	C29—C20—C21	108.5 (3)
H7A—C7—H7B	107.6	C30—C20—C19	110.7 (3)
C7—C8—C26	105.8 (3)	C29—C20—C19	109.0 (3)
C7—C8—C9	109.5 (2)	C21—C20—C19	108.2 (2)
C26—C8—C9	111.4 (3)	C18—C19—C20	113.8 (3)
C7—C8—C14	109.8 (3)	C18—C19—H19A	108.8
C26—C8—C14	112.4 (2)	C20—C19—H19A	108.8
C9—C8—C14	108.0 (2)	C18—C19—H19B	108.8
C8—C26—H26A	109.5	C20—C19—H19B	108.8
C8—C26—H26B	109.5	H19A—C19—H19B	107.7
H26A—C26—H26B	109.5	C17—C18—C19	111.9 (2)
C8—C26—H26C	109.5	C17—C18—C13	99.7 (2)
H26A—C26—H26C	109.5	C19—C18—C13	114.1 (2)
H26B—C26—H26C	109.5	C17—C18—H18	110.2
C11—C9—C8	109.2 (2)	C19—C18—H18	110.2
C11—C9—C10	114.1 (2)	C13—C18—H18	110.2
C8—C9—C10	117.6 (2)	C20—C29—H29A	109.5
C11—C9—H9	104.9	C20—C29—H29B	109.5
C8—C9—H9	104.9	H29A—C29—H29B	109.5
C10—C9—H9	104.9	C20—C29—H29C	109.5
C1—C10—C25	107.7 (3)	H29A—C29—H29C	109.5
C1—C10—C5	107.4 (2)	H29B—C29—H29C	109.5
C25—C10—C5	114.4 (3)	C20—C30—H30A	109.5
C1—C10—C9	107.8 (2)	C20—C30—H30B	109.5
C25—C10—C9	113.3 (2)	H30A—C30—H30B	109.5
C5—C10—C9	106.0 (2)	C20—C30—H30C	109.5
C12—C11—C9	112.4 (3)	H30A—C30—H30C	109.5
C12—C11—H11A	109.1	H30B—C30—H30C	109.5
C9—C11—H11A	109.1	O28—C28—O13	121.1 (3)
C12—C11—H11B	109.1	O28—C28—C17	129.2 (3)
C9—C11—H11B	109.1	O13—C28—C17	109.6 (2)
H11A—C11—H11B	107.9	C10—C25—H25A	109.5
C13—C12—C11	115.7 (2)	C10—C25—H25B	109.5
C13—C12—H12A	108.4	H25A—C25—H25B	109.5
C11—C12—H12A	108.4	C10—C25—H25C	109.5
C13—C12—H12B	108.4	H25A—C25—H25C	109.5
C11—C12—H12B	108.4	H25B—C25—H25C	109.5
H12A—C12—H12B	107.4		
			
C10—C1—C2—C3	−54.8 (4)	O13—C13—C14—C27	−168.8 (2)
C1—C2—C3—O3	−128.1 (4)	C12—C13—C14—C27	72.3 (3)
C1—C2—C3—C4	51.3 (5)	C18—C13—C14—C27	−58.8 (3)
O3—C3—C4—C23	−102.1 (4)	O13—C13—C14—C8	69.8 (3)
C2—C3—C4—C23	78.4 (4)	C12—C13—C14—C8	−49.1 (3)
O3—C3—C4—C24	15.4 (5)	C18—C13—C14—C8	179.8 (2)
C2—C3—C4—C24	−164.1 (3)	C7—C8—C14—C15	−61.7 (3)
O3—C3—C4—C5	133.0 (4)	C26—C8—C14—C15	55.7 (4)
C2—C3—C4—C5	−46.5 (4)	C9—C8—C14—C15	179.0 (3)
C3—C4—C5—C6	179.5 (3)	C7—C8—C14—C27	58.0 (3)
C23—C4—C5—C6	57.9 (5)	C26—C8—C14—C27	175.4 (3)
C24—C4—C5—C6	−63.5 (4)	C9—C8—C14—C27	−61.4 (3)
C3—C4—C5—C10	47.6 (4)	C7—C8—C14—C13	177.2 (2)
C23—C4—C5—C10	−74.0 (4)	C26—C8—C14—C13	−65.4 (3)
C24—C4—C5—C10	164.6 (3)	C9—C8—C14—C13	57.8 (3)
C10—C5—C6—C7	−63.9 (4)	C27—C14—C15—C16	74.8 (4)
C4—C5—C6—C7	160.9 (3)	C13—C14—C15—C16	−41.1 (4)
C5—C6—C7—C8	56.5 (4)	C8—C14—C15—C16	−163.6 (3)
C6—C7—C8—C26	74.0 (4)	C14—C15—C16—C17	44.9 (4)
C6—C7—C8—C9	−46.1 (4)	C15—C16—C17—C28	45.6 (4)
C6—C7—C8—C14	−164.5 (3)	C15—C16—C17—C18	−61.7 (4)
C7—C8—C9—C11	178.4 (3)	C15—C16—C17—C22	169.5 (3)
C26—C8—C9—C11	61.8 (3)	C28—C17—C22—C21	−70.9 (4)
C14—C8—C9—C11	−62.0 (3)	C18—C17—C22—C21	43.2 (4)
C7—C8—C9—C10	46.4 (3)	C16—C17—C22—C21	167.7 (3)
C26—C8—C9—C10	−70.2 (3)	C17—C22—C21—C20	−51.3 (4)
C14—C8—C9—C10	166.0 (2)	C22—C21—C20—C30	−63.9 (4)
C2—C1—C10—C25	−70.1 (4)	C22—C21—C20—C29	176.0 (3)
C2—C1—C10—C5	53.6 (4)	C22—C21—C20—C19	57.8 (4)
C2—C1—C10—C9	167.3 (3)	C30—C20—C19—C18	64.2 (3)
C6—C5—C10—C1	174.8 (3)	C29—C20—C19—C18	−175.6 (3)
C4—C5—C10—C1	−51.7 (3)	C21—C20—C19—C18	−57.7 (3)
C6—C5—C10—C25	−65.7 (4)	C28—C17—C18—C19	78.8 (3)
C4—C5—C10—C25	67.8 (4)	C22—C17—C18—C19	−42.3 (4)
C6—C5—C10—C9	59.8 (3)	C16—C17—C18—C19	−168.3 (3)
C4—C5—C10—C9	−166.7 (2)	C28—C17—C18—C13	−42.3 (3)
C11—C9—C10—C1	62.4 (3)	C22—C17—C18—C13	−163.4 (3)
C8—C9—C10—C1	−167.9 (3)	C16—C17—C18—C13	70.7 (3)
C11—C9—C10—C25	−56.7 (4)	C20—C19—C18—C17	50.4 (3)
C8—C9—C10—C25	73.1 (4)	C20—C19—C18—C13	162.7 (2)
C11—C9—C10—C5	177.1 (2)	O13—C13—C18—C17	43.2 (3)
C8—C9—C10—C5	−53.2 (3)	C12—C13—C18—C17	158.1 (2)
C8—C9—C11—C12	58.6 (3)	C14—C13—C18—C17	−71.6 (3)
C10—C9—C11—C12	−167.6 (2)	O13—C13—C18—C19	−76.3 (3)
C9—C11—C12—C13	−50.4 (3)	C12—C13—C18—C19	38.7 (3)
C28—O13—C13—C12	−147.8 (2)	C14—C13—C18—C19	169.0 (2)
C28—O13—C13—C18	−27.9 (3)	C13—O13—C28—O28	−179.1 (3)
C28—O13—C13—C14	90.2 (3)	C13—O13—C28—C17	0.6 (3)
C11—C12—C13—O13	−73.7 (3)	C18—C17—C28—O28	−153.0 (4)
C11—C12—C13—C18	175.6 (2)	C22—C17—C28—O28	−29.3 (5)
C11—C12—C13—C14	45.4 (3)	C16—C17—C28—O28	93.8 (4)
O13—C13—C14—C15	−52.4 (3)	C18—C17—C28—O13	27.3 (3)
C12—C13—C14—C15	−171.3 (3)	C22—C17—C28—O13	151.1 (3)
C18—C13—C14—C15	57.6 (3)	C16—C17—C28—O13	−85.9 (3)

### Hydrogen-bond geometry (Å, °)

**Table:**

*D*—H···*A*	*D*—H	H···*A*	*D*···*A*	*D*—H···*A*
C26—H26B···O13	0.96	2.40	2.865 (4)	109
